# Automatic measure and normalization of spinal cord cross-sectional area using the pontomedullary junction

**DOI:** 10.3389/fnimg.2022.1031253

**Published:** 2022-11-02

**Authors:** Sandrine Bédard, Julien Cohen-Adad

**Affiliations:** ^1^NeuroPoly Lab, Institute of Biomedical Engineering, Polytechnique Montreal, Montreal, QC, Canada; ^2^Functional Neuroimaging Unit, Centre de recherche de l'Institut universitaire de gériatrie de Montréal (CRIUGM), University of Montreal, Montreal, QC, Canada; ^3^Mila - Quebec AI Institute, Montreal, QC, Canada

**Keywords:** spinal cord, MRI, normalization, inter-subject variability, biomarker

## Abstract

Spinal cord cross-sectional area (CSA) is a relevant biomarker to assess spinal cord atrophy in neurodegenerative diseases. However, the considerable inter-subject variability among healthy participants currently limits its usage. Previous studies explored factors contributing to the variability, yet the normalization models required manual intervention and used vertebral levels as a reference, which is an imprecise prediction of the spinal levels. In this study we implemented a method to measure CSA automatically from a spatial reference based on the central nervous system (the pontomedullary junction, PMJ), we investigated factors to explain variability, and developed normalization strategies on a large cohort (*N* = 804). Following automatic spinal cord segmentation, vertebral labeling and PMJ labeling, the spinal cord CSA was computed on T1w MRI scans from the UK Biobank database. The CSA was computed using two methods. For the first method, the CSA was computed at the level of the C2–C3 intervertebral disc. For the second method, the CSA was computed at 64 mm caudally from the PMJ, this distance corresponding to the average distance between the PMJ and the C2–C3 disc across all participants. The effect of various demographic and anatomical factors was explored, and a stepwise regression found significant predictors; the coefficients of the best fit model were used to normalize CSA. CSA measured at C2–C3 disc and using the PMJ differed significantly (paired *t*-test, *p*-value = 0.0002). The best normalization model included thalamus, brain volume, sex and the interaction between brain volume and sex. The coefficient of variation went down for PMJ CSA from 10.09 (without normalization) to 8.59%, a reduction of 14.85%. For CSA at C2–C3, it went down from 9.96 to 8.42%, a reduction of 15.13 %. This study introduces an end-to-end automatic pipeline to measure and normalize cord CSA from a neurological reference. This approach requires further validation to assess atrophy in longitudinal studies. The inter-subject variability of CSA can be partly accounted for by demographics and anatomical factors.

## Introduction

Various neurodegenerative diseases such as multiple sclerosis (MS) are associated with spinal cord (SC) atrophy, which is caused by demyelination, neuronal and/or axonal loss (Lukas et al., 2013; Bonacchi et al., 2020). New techniques have now become available through recent advancement in magnetic resonance imaging (MRI) and are relevant to assess SC atrophy (Moccia et al., 2019).

SC atrophy at the upper cervical levels can be defined within its cross-sectional area (CSA) (Losseff et al., 1996; Lukas et al., 2013). The use of this metric is yet still limited due to considerable inter-subject variability. Finding factors that contribute to the observed variability is important to improve the SC CSA's sensitivity and specificity.

Various studies have explored the correlation between SC CSA and demographic, anatomical and biological factors. Sex was a relevant factor to explain SC CSA variability, with females having significantly smaller SC CSA than males (Engl et al., 2013; Papinutto et al., 2020; Solstrand Dahlberg et al., 2020). While the majority of studies have reported this significant effect, Fradet et al. (2014) found it to be an irrelevant factor and Papinutto et al. (2015) did observe the trend, but no statistical difference was found. However, the absence of a statistical difference could be explained by the relatively small sample size (30 participants).

Regarding the effect of age, a decrease of SC CSA was previously reported (Ishikawa et al., 2003; Kato et al., 2012; Engl et al., 2013; Papinutto et al., 2015, 2020). However, the effect was not significant (Engl et al., 2013; Papinutto et al., 2015, 2020). This trend is accentuated for older populations, but the effect of age is still small (Engl et al., 2013). An increase of SC CSA values followed by a decrease at 45 years old was also reported, but the effect was not significant (Papinutto et al., 2020). The effect of age on SC CSA needs further investigation, small sample size and narrow range of age are limiting factors to assess the effect of age on SC CSA.

As for height and body weight, no significant effect on SC CSA was found according to recent studies (Papinutto et al., 2015, 2020; Solstrand Dahlberg et al., 2020). The effect of height may be driven by sex differences (Papinutto et al., 2020). Body mass index (BMI) was also tested as a normalization strategy for SC volume, but results were inconclusive as inter-subject variability was increased (Sanfilipo et al., 2004). In another study, no correlation was found with BMI by Solstrand Dahlberg et al. (2020).

Strong correlation between brain metrics and SC CSA were reported in prior studies (Engl et al., 2013; Papinutto et al., 2015, 2020; Solstrand Dahlberg et al., 2020). White matter (WM) volume significantly explains upper cervical area variability as opposed to cerebrospinal fluid (CSF) volume, which was not significant according to Engl et al. (2013). In addition, brain volume correlated strongly with SC CSA as for intracranial volume (Papinutto et al., 2015; Solstrand Dahlberg et al., 2020). Papinutto et al. (2020) also found this effect with Vscale (scaling factor for head size normalization) and considered it as the most promising factor for normalization strategies. Intracranial volume was also considered for normalization of SC volume but had limited utility since it generally diminished the ability to detect clinical-radiological correlations (Healy et al., 2012; Oh et al., 2014). Since SC CSA is a useful metric to assess SC atrophy and brain volume changes have also been associated with those pathologies, intracranial volume would be a better factor to consider for normalization strategies (Kesenheimer et al., 2021). A strong correlation was also found between thalamus volume and SC CSA by Solstrand Dahlberg et al. (2020). Axial canal area was also a significant factor and promising for normalization strategies but has not been explored yet by many (Papinutto et al., 2020; Kesenheimer et al., 2021). A notable difficulty for computing the axial canal area is the ability to properly segment it.

Only a few of the previous cited works have explored normalization strategies for SC CSA. SC length was a relevant factor for SC volume normalization compared to intracranial volume (Healy et al., 2012; Oh et al., 2014). Mean SC volume in healthy participants was also used to normalize SC volume of patients with MS (Ruggieri et al., 2021). Regarding SC CSA, age, intracranial volume, and sagittal vertebral area were the most promising independent variables found by Papinutto et al. (2015), but the method was based on 30 participants only. Another model later including Vscale, axial canal product (product of maximum axial anterior-posterior and lateral diameters of the cervical SC) based on 129 participants significantly reduced SC CSA variability (Papinutto et al., 2020). Brain WM volume, sex and spinal canal area formed a relevant normalization strategy. Total intracranial volume can also replace brain WM volume for subjects with diseases affecting WM. The main limitation here was the relatively small number of participants (*N* = 61) (Kesenheimer et al., 2021). As we can observe, brain/skull metrics and sex are important factors to consider in a possible normalization method. Also, the mentioned normalization methods are only reported in the related papers; they are not easily reusable to integrate directly within analysis pipelines.

In addition to the biological-derived normalization strategy, previous studies have reported a variability in CSA measures associated with the MRI acquisition parameters, and segmentation method (Kearney et al., 2014; Papinutto and Henry, 2019; Chien et al., 2020; Cohen-Adad et al., 2021).

The majority of the studies regarding SC CSA use vertebral levels as an anatomical reference (Casserly et al., 2018; Moccia et al., 2019). However, inferring the position of the spinal segments using vertebral levels is imprecise, adding variability to CSA measures (Cadotte et al., 2015). Inferring neuroanatomic positions with vertebral bodies doesn't consider neck flexion and extension. In addition, because of the intrinsic inter-subject variability, the vertebral bodies only give a rough approximation of the spinal segments (Cadotte et al., 2015). Segmental nerve rootlets would provide a proper identification of the spinal segments, it is however difficult to identify and requires high resolution T2w scans and an expert rater to identify them. A few studies have attempted to bypass the vertebral-based limitation using the distance from an anatomical landmark, such as the pontomedullary junction (PMJ) (Stroman et al., 2008; Cadotte et al., 2015; Amann et al., 2016), and the conus medullaris (Tsagkas et al., 2018). However, these methods require manual intervention limiting large-scale applications. In addition, the PMJ was not used in the context of measuring SC CSA (Stroman et al., 2008; Cadotte et al., 2015; Amann et al., 2016; Tsagkas et al., 2018). For cervical SC measures, the PMJ is a more appropriate landmark due to its proximity to the cervical SC, hence limiting the required field of view in MRI scans, compared to conus medullaris which could be more appropriate for lumbar SC measures.

While SC CSA variability across participants was shown to be associated with multiple demographic, anatomical and biological factors (Papinutto et al., 2015, 2020; Solstrand Dahlberg et al., 2020; Kesenheimer et al., 2021), no previous studies have addressed the variability associated with limitation of vertebral based SC CSA measurement in the search for a normalization method of SC CSA at a large scale.

In this study we quantify the contribution of various factors on the inter-subject variability in cervical SC CSA measurements. We notably introduce a method to replace the commonly used vertebral-based referencial system (Moccia et al., 2019) by an anatomical reference from the central nervous system to overcome the imprecise prediction of the spinal segments. More precisely, we (1) establish a fully-automatic MRI data processing pipeline to compute SC CSA, (2) process MRI data from a subset (*N* = 804) of the UK Biobank database, (3) introduce a method to automatically use the PMJ as a referential system to measure SC CSA, (4) develop a statistical model and normalization method for SC CSA measurements.

## Materials and methods

### Demography

1,000 participants (48–80 years old, 56.3% female) were selected from the UK Biobank database. Not knowing the effect size we were after, we could not base this number on any reliable power analysis. Hence, the number of participants was selected as a compromise between the statistical power we wanted to achieve in comparison with the previously-published studies addressing similar scientific questions (typically <300 participants) and the time required to manually validate each step of the processing pipeline (visual inspection, manual correction of SC segmentation and/or PMJ labeling and/or vertebral labeling).

Participants with a history of neurological diseases were excluded from the study. Fields from the UK Biobank dataset included in the category *Nervous system disorders*^1^ were used to identify these participants. This brought the number of participants from 1,000 to 972.

### Image acquisition

Data used for this study were unprocessed NIfTI T1w structural scans from the UK Biobank Brain Imaging dataset (Miller et al., 2016). Images were acquired in four different assessment centers on a Siemens Skyra 3T running VD13A SP4 with a standard Siemens 32-channel receive head coil. T1w structural scan has a field of view of 208 × 256 × 256 with an isotropic resolution of 1 mm^3^. The superior-inferior field of view of 256 mm typically covers down to C3 vertebral level, which is relevant for the present study as SC CSA was measured around the C2–C3 vertebral level. The UK Biobank data includes preprocessed data (corrected for gradient non-linearity and masked), however we could not use these data because the SC was masked out. We therefore used the unprocessed T1w images as input of the processing pipeline described in the next section.

### Data processing

Data processing pipeline is based on spine-generic v2.6 pipeline^2^ (Cohen-Adad et al., 2021) and SCT v5.4^3^ (De Leener et al., 2017). The processing pipeline and its documentation are both available on GitHub.^4^

**Figure 1:**
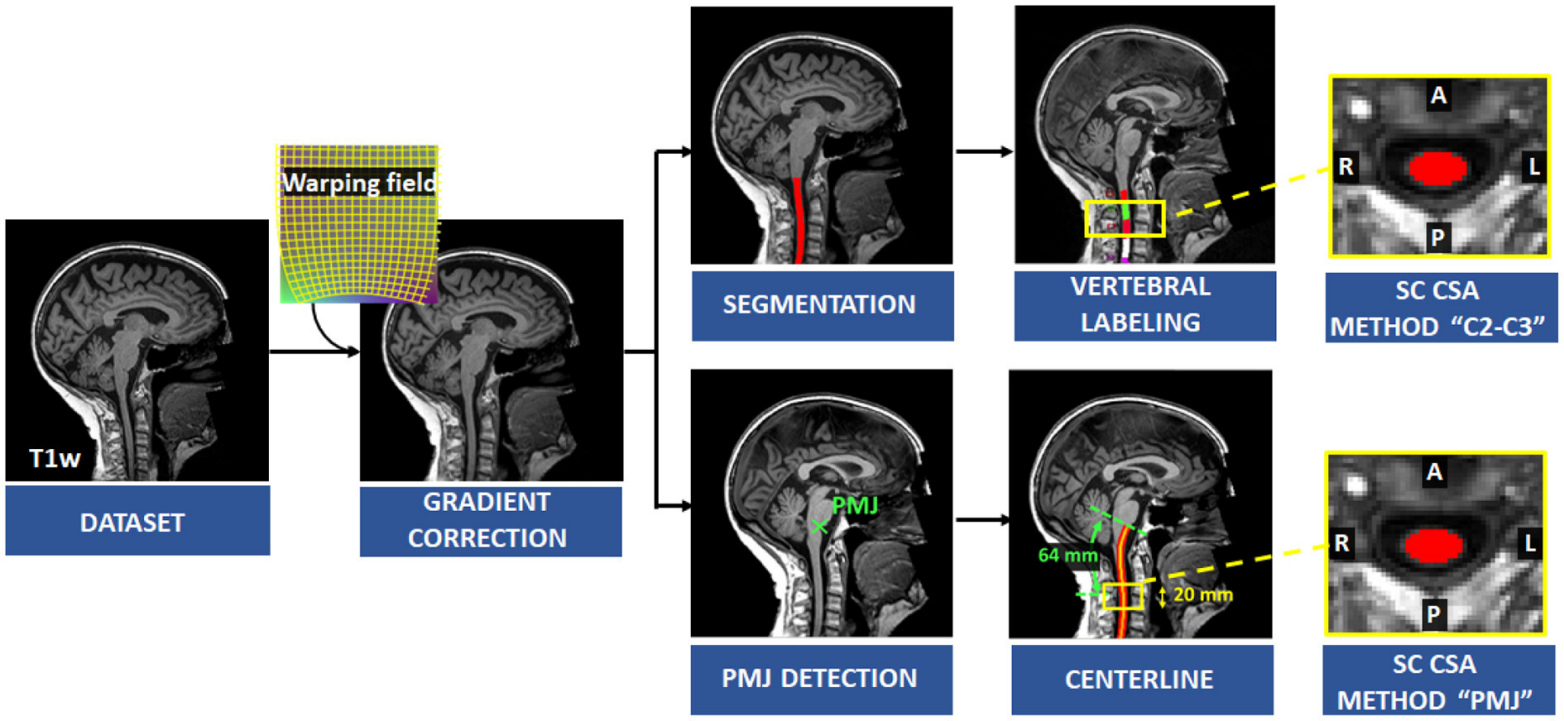
Overview of the processing pipeline. Correction for gradient non-linearities was applied on the T1w image and SC was segmented automatically. Vertebral levels were identified, and SC CSA was computed at C2–C3 levels. The PMJ was labeled, and the centerline was extracted to compute SC CSA from the PMJ.

Figure 1 presents an overview of the processing pipeline. First, all images were reoriented to right-inferior-posterior orientation. Since SC CSA is computed using T1w brain images and SC is in the periphery of the images' field of view, gradient non-linearity distortions have a considerable effect on CSA measures, depending on participant positioning (Papinutto et al., 2018). Gradient non-linearity correction was applied by the UK Biobank after brain extraction and was therefore not possible for us to use since the cervical spine is the subject of investigation in this study. Thus, correction for gradient non-linearities was applied to the unprocessed images using *gradunwrap* from the HCP project (Glasser et al., 2013) and the coefficient file for the Siemens Skyra 3T gradient system. Then, the SC was segmented automatically using SCT's sct_deepseg_sc (Gros et al., 2019). SC CSA was computed using SCT's sct_process_segmentation with two different methods further explained below.

#### CSA based on distance from neurological reference: Pontomedullary junction

To overcome the limitation of SC segment prediction with vertebral bodies, SC CSA was measured from a distance of a neurological reference; we chose the PMJ (Stroman et al., 2008; Cadotte et al., 2015).

First, the PMJ was identified using SCT'S sct_detect_pmj. Briefly, a 2D support vector machine trained with histogram of oriented gradient features (HOG + SVM 2D classifier) was run on the mid-sagittal slice to detect the PMJ (Gros et al., 2017). However, the mid-sagittal slice does not necessarily correspond to the anatomical medial plane if the participant's head is slightly tilted in the scanner for example. We used a sliding window centered on the first estimated PMJ coordinate based on the mid-sagittal plane and computed cross-correlation within the window and its mirror image in the right-left orientation. We assumed that the maximum cross-correlation corresponds to the right-left symmetry slice. The HOG + SVM 2D classifier was run again on the updated medial plane.

**Figure 2:**
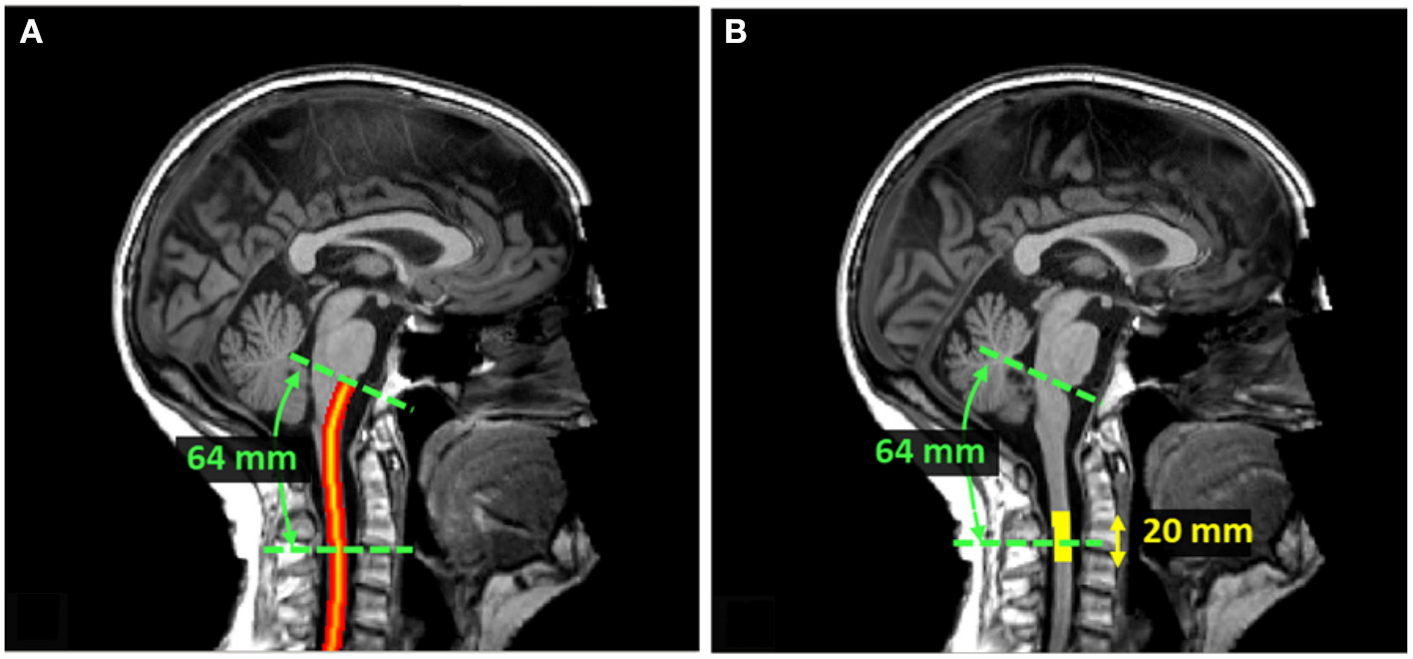
**(A)** SC centerline from PMJ. The SC centerline was extracted from SC segmentation and the PMJ label using linear interpolation and smoothing. The distance from PMJ is measured along the centerline following the arc-length. **(B)** Extent mask to average SC CSA. At 64 mm from PMJ, CSA was computed slice-wise, corrected for angulation, and averaged within a 20 mm extent. The extent mask is centered at 64 mm from PMJ.

Since the SC curvature associated with cervical lordosis varies across individuals, the distance from the PMJ was computed along the SC centerline following the arc-length. SC segmentation normally doesn't go as high as the PMJ. The PMJ coordinate was then added to the SC segmentation prior to extracting the SC centerline. Linear interpolation and smoothing were used to extract the SC centerline. SC CSA was computed at the mean distance between the C2–C3 disc and the PMJ across all participants, which corresponds to 64 mm. SC CSA was computed along the SC centerline and then averaged on a 20 mm extent as presented in Figure 2.

#### CSA based on C2–C3 vertebral levels

The second and more commonly used method to compute the SC CSA was to use C2–C3 vertebral levels as an anatomical reference for spinal segments (Casserly et al., 2018; Moccia et al., 2019). We proceeded to vertebral labeling using sct_label_vertebrae (Ullmann et al., 2014). SC CSA was then averaged across C2–C3.

### Quality control

After running a first pass of the automatic analysis pipeline, we inspected results of the SC segmentations and of the PMJ and vertebral labeling using SCT's quality report (sct_qc). For more details about the procedure, see: https://github.com/sct-pipeline/ukbiobank-spinalcord-csa/blob/v1.1/README.md#quality-control. Segmentation and labeling errors typically occurred if there were important artifacts in the image, such as ghosting caused by poor shimming and/or participant motion (e.g., swallowing, head tilting). PMJ and vertebral labeling errors also sometimes happened if the field of view was improperly positioned (e.g., excessive rotation about the right-left axis). If images were too artefacted to produce reliable morphometric measures (see examples at https://github.com/sct-pipeline/ukbiobank-spinalcord-csa/issues/61), these images were excluded from the statistical analysis. This brought the number of participants from 972 to 826.

For less severe artifacts, manual corrections were done on the segmentation and/or labeling. Making these corrections ensured that the derived CSA measures are reliable. Segmentations were corrected using ITK-SNAP by adding or removing voxels when appropriate. To correct vertebral labeling, we manually labeled the posterior tip of the intervertebral discs C1–C2, C2–C3 and C3–C4 using SCT's sct_label_utils. A similar approach was done for the PMJ. All manual corrections were added to the dataset under the folder “/derivatives/labels” that follows the BIDS convention. The whole analysis was then re-run and when existing, the corrected segmentations/labels were used in lieu of the automatic segmentations/labels.

Processing was distributed across 40 CPU cores (one participant per CPU core) using sct_run_batch on a 64-core CPU cluster. Processing took 01 h 53 m 59 s.

### Statistical analyses

#### Comparison of CSA measure with both methods

To study the relationship between the PMJ-based and vertebral-based CSA measures, we built a scatterplot and derived a model. We also computed the distance between the C2–C3 disc and PMJ. Mean, standard deviation (STD), median and coefficient of variation (COV) for both CSA measures were computed. We performed a two-sided paired *T*-test to assess if there is a statistically significant difference between PMJ-based CSA vs. C2–C3-based CSA. All subsequent analyses were done on both CSA at 64 mm from the PMJ and at the C2–C3 disc.

#### Correlations with physical and brain measures

In this study, the effect of sex, age, physical measures, and brain measures on SC CSA was explored by calculating *Pearson's* correlation coefficients. Physical measures included height and weight. Brain measures included brain WM volume, brain gray matter (GM) volume, brain volume, brain volume normalized for head size, thalamus volume and ventricular CSF volume. All data (images and demographic measures) were acquired at the same time for each participant and were available in the UK Biobank database. The brain metrics were computed by the UK Biobank team as part of their processing pipeline (Alfaro-Almagro et al., 2018).

#### Effect of sex and age

To assess the effect of sex on SC CSA, a *T*-test for two independent samples was performed to establish if the mean CSA for male and female has a significant difference. We fitted a linear and quadratic regression to assess the effect of age on SC CSA. *R*^2^ was reported.

#### Multilinear regression

The effect of all candidate predictors was evaluated using a multilinear analysis. Sex was included as a dichotomous variable to the regression. Any participant missing a parameter was excluded from this analysis. This brought the number of participants from 826 to 804. To select the relevant predictors of the multilinear regression, a stepwise method was used. The predictors are added to the model from the highest correlation with CSA to the lowest if they are significant (*p*-value < 0.05). After each addition of predictors, the significance of the current parameters was computed again, and parameters with a *p*-value > 0.05 were excluded from the model (Toutenburg et al., 1969). The level of significance was the same for both entry and exit tests. To validate the model, we proceeded to a residual analysis and computed *R*^2^.

*Pearson's* correlation coefficient between candidate predictors was used to choose which parameter to include in the stepwise model due to possible collinearity between parameters.

#### Normalization method

With the best multilinear regression fit, a regression-based residual method (Sanfilipo et al., 2004; Papinutto et al., 2020) was developed using the significant predictors, as described in the following equation:


$$\begin{array}{l} CSA_{norm}^{i}\;=CSA_{meas}^{i}\;+c_{1}\left(X_{{1,\;mean}_{\;}}-X_{1,\;meas}^{\;i}\right)\;\\ +c_{2}\left(X_{{2,\;mean}_{\;}}-{X_{2,\;meas}^{\;i}}_{\;}\right)+...+c_{n}\left(X_{{n,\;mean}_{\;}}-X_{n,\;meas}^{\;i}\right) \end{array}$$

where $CSA_{meas}^{i}$ is the computed SC CSA value from a given participant *i*, $CSA_{norm}^{i}$ is the normalized CSA value, *c*_*j*_ are the coefficients of the multilinear regression, $X_{j,\;mean}^{\;}$ are the mean values of all significant predictors, $X_{j,\;meas}^{i}$ are values for the given participant's predictors, for *j* predictors (Papinutto et al., 2020).

Since this method assumes that the regression line slopes are parallel for both groups for sex (Sanfilipo et al., 2004), the interaction between significant predictors and sex was also explored afterward. If the interaction term was significant, it was added to the model. The interaction term corresponds to the predictor multiplied by sex (0 or 1) as we can see in the following equation:


$$\begin{array}{l} y\;=\;c_{1}\;*\;X_{1}\;+\;c_{2\;}*\;sex\;+\;c_{3\;}*\;X_{1}*\;sex\; \end{array}$$

The effect of normalization was then evaluated by comparing the COV of the normalized SC CSA ($CSA_{norm}^{i}$) and the measured CSA ($CSA_{meas}^{i}$). We also present the mean (μ_*norm*_) and STD (σ_*norm*_) of the normalized CSA measures ($CSA_{norm}^{i}$) and the *z*-score equation:


$$\begin{array}{l} z\text{-score}=\frac{CSA_{norm}-\mu_{norm}}{\sigma_{norm}} \end{array}$$

## Results

### SC CSA

In this study we compared SC CSA measured using the PMJ as a reference or the C2–C3 disc as a reference (more popular). When using the PMJ as a reference (64 mm caudal to the PMJ), the CSA ranged between 51.9 and 95.6 mm^2^ (mean ± STD: 66.2 ± 6.69 mm^2^). The COV was 10.09%. When using C2–C3 vertebral levels as a reference, the CSA ranged between 51.5 and 96.9 mm^2^ (mean ± STD: 66.4 ± 6.61. mm^2^). The COV was 9.96 %.

**Figure 3:**
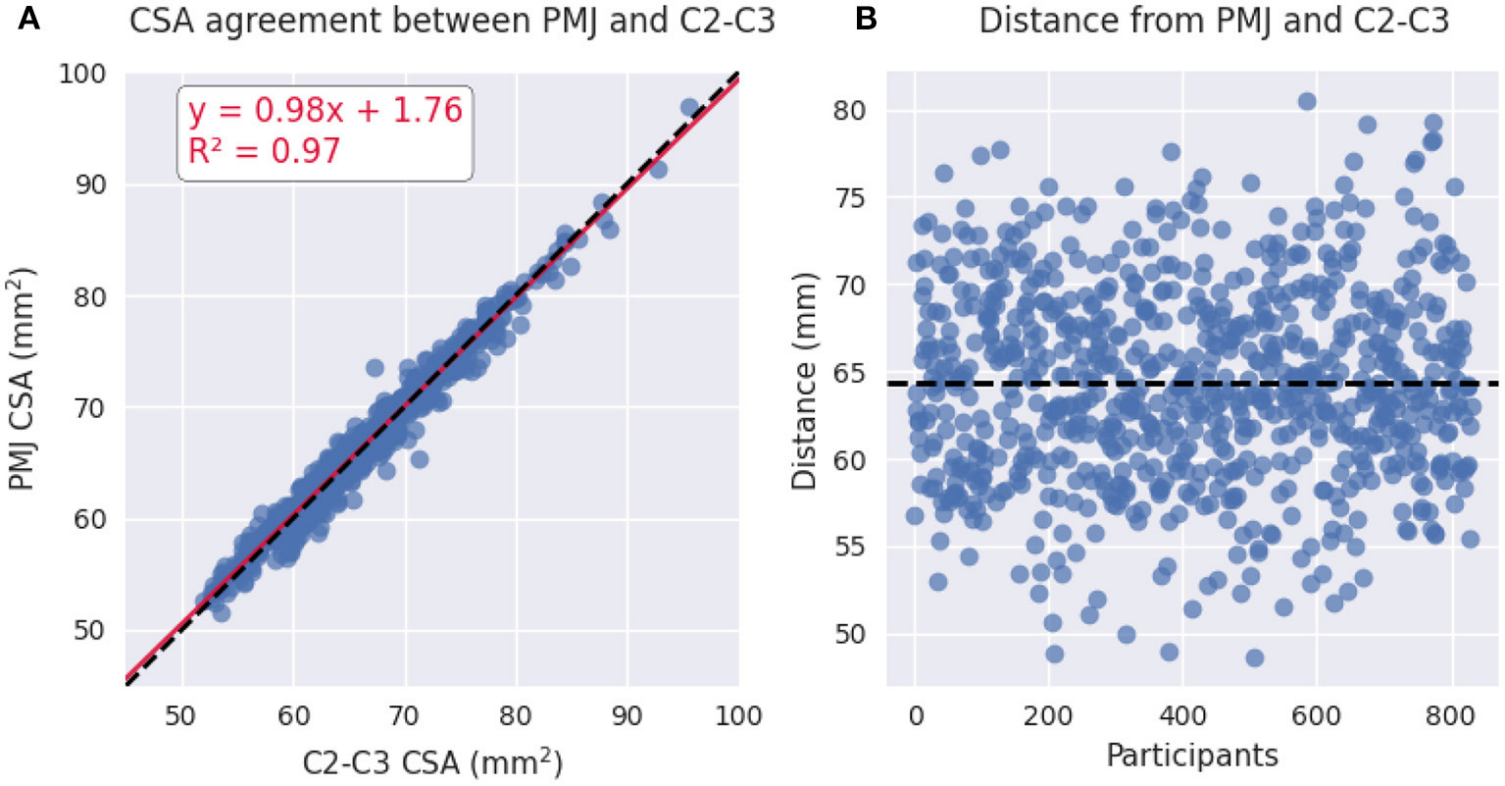
**(A)** Scatterplot of PMJ-based CSA at 64 mm and vertebral-based CSA at C2–C3 vertebral levels. **(B)** Scatterplot of the distance between the PMJ and the C2–C3 disc.

Figure 3A shows the relationship between CSA at 64 mm from the PMJ and CSA at C2–C3 vertebral levels. The linear regression led to a *R*^2^ of 0.97. Figure 3B shows a scatterplot of the distance from the PMJ and C2–C3 disc. Mean distance from the PMJ to the C2–C3 disc is 64.37 ± 5.53 mm.

We found a significant difference between the CSA calculated at 64 mm from the PMJ and the CSA measured at the C2–C3 disc (paired *t*-test, *t* = −3.71, *p*-value = 0.0002).

### Statistical analyses

#### Correlations with physical and brain measures

We investigated the relationship of SC CSA with sex, age, physical and brain measures. Results of the correlation analysis (*Pearson's*) are reported in Table 1. Note that this correlation matrix is not corrected for multiple comparisons because its purpose was only to explore existing correlations. In subsequent analysis (see *Multilinear regression*), a multivariate analysis will account for the number of regressors in the estimated *p*-values. Scatterplots of CSA(PMJ) and all parameters are shown in Supplementary material (S1–S8). Ventricular CSF volume was the only parameter to present a non-significant correlation coefficient with both SC CSA measures (*p*-value > 0.05). Thalamus, brain, brain WM and brain GM volume present the highest correlations out of all parameters (*Pearson's r* > 0.4). Among parameters, we notice in particular a very strong correlation between thalamus volume and brain, brain WM and brain GM volumes.

**Table 1:** Pearson's correlation among SC CSA, physical, and brain measures.

	**Sex**	**Age**	**Height**	**Weight**	**Vscale**	**Ventricular CSF volume**	**Brain GM volume**	**Brain WM volume**	**Brain volume norm**	**Brain volume**	**Thalamus volume**	**CSA(PMJ)**	**CSA(C2–C3)**
Sex	1.0	0.07*	0.71***	0.48***	−0.59***	0.33***	0.39***	0.53***	−0.2***	0.49***	0.36***	0.19***	0.18***
Age		1.0	−0.07*	−0.05	−0.05	0.41***	−0.35***	−0.12***	−0.56***	−0.24***	−0.33***	−0.18***	−0.18***
Height			1.0	0.57***	−0.58***	0.21***	0.45***	0.51***	−0.13***	0.51***	0.42***	0.2***	0.19***
Weight				1.0	−0.41***	0.16***	0.3***	0.39***	−0.09**	0.36***	0.25***	0.13***	0.12***
Vscale					1.0	−0.46***	−0.78***	−0.84***	0.24***	−0.85***	−0.61***	−0.32***	−0.32***
Ventricular CSF volume						1.0	0.08*	0.25***	−0.53***	0.18***	−0.09**	−0.02	−0.04
Brain GM volume							1.0	0.81***	0.33***	0.94***	0.75***	0.4***	0.4***
Brain WM volume								1.0	0.22***	0.96***	0.76***	0.45***	0.46***
Brain volume norm									1.0	0.29***	0.36***	0.24***	0.25***
Brain volume										1.0	0.79***	0.45***	0.45***
Thalamus volume											1.0	0.51***	0.52***
CSA(PMJ)												1.0	0.99***
CSA(C2–C3)													1.0

*p*-value: ^*^0.01 < *P* < 0.05, ^**^0.001 < *P* < 0.01, ^***^P < 0.001.

#### Effect of sex and age

**Figure 4:**
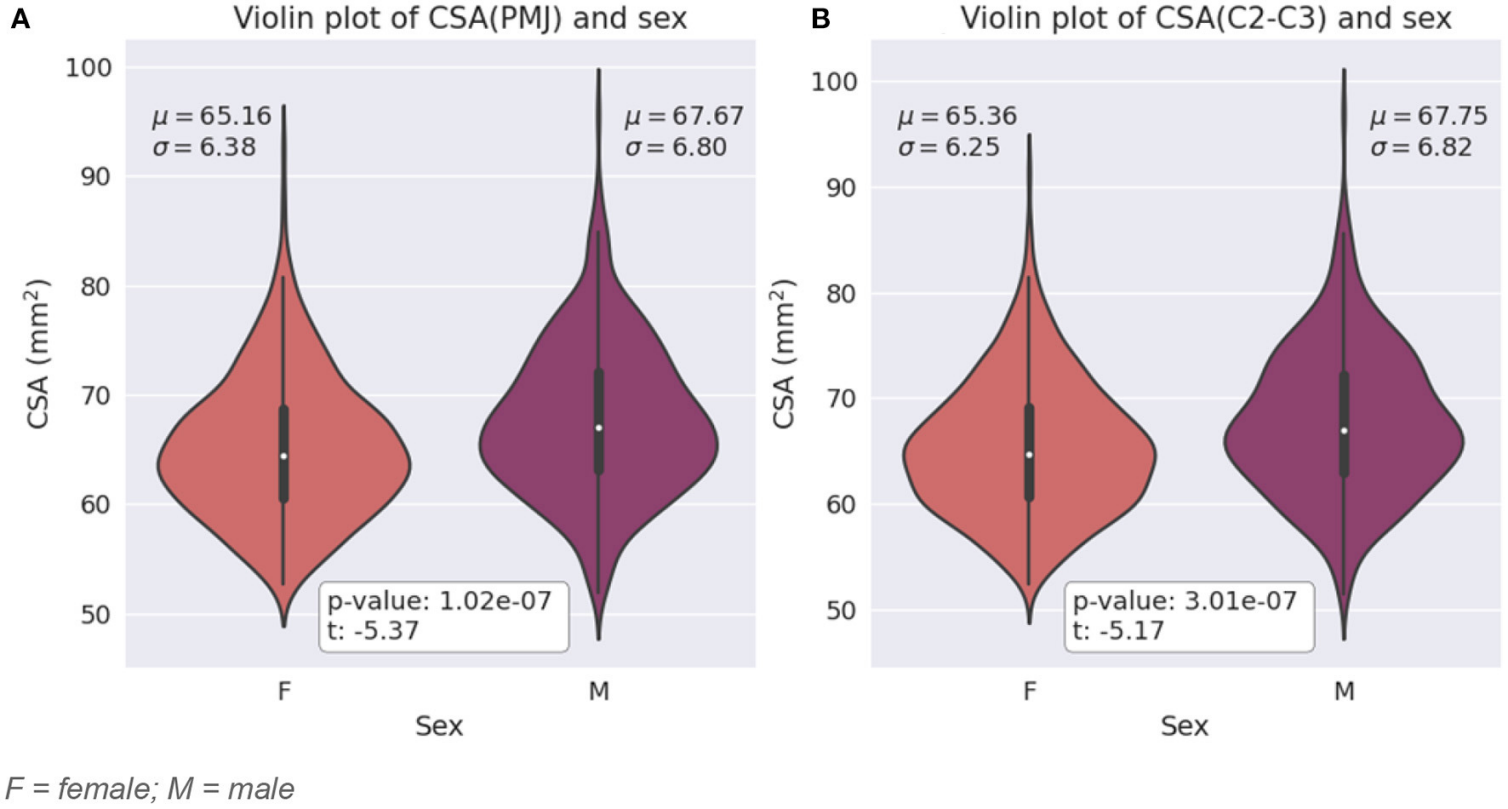
**(A)** Violin plot of SC CSA at 64 mm from the PMJ for female and male with mean (μ) and standard deviation (σ) for each sex and *t*-value and *p*-value from the two independent samples *t*-test. **(B)** Violin plot of SC CSA at C2–C3 disc for female and male with mean (μ) and standard deviation (σ) for each sex *t*-value and *p*-value from the two independent samples *T*-test. F = female; M = male.

Participants in this study include 43.7% of males and 56.3% of females. Figure 4 presents SC CSA violin plots for female and male with mean and STD. We found a significant difference for CSA between female and male for both CSA at 64 mm from the PMJ and at C2–C3 disc (**CSA(PMJ)**: *t* = −5.37, *p*-value < 10^−7^, **CSA(C2–C3)**: *t* = −5.17, *p*-value < 10^−7^).

**Figure 5:**
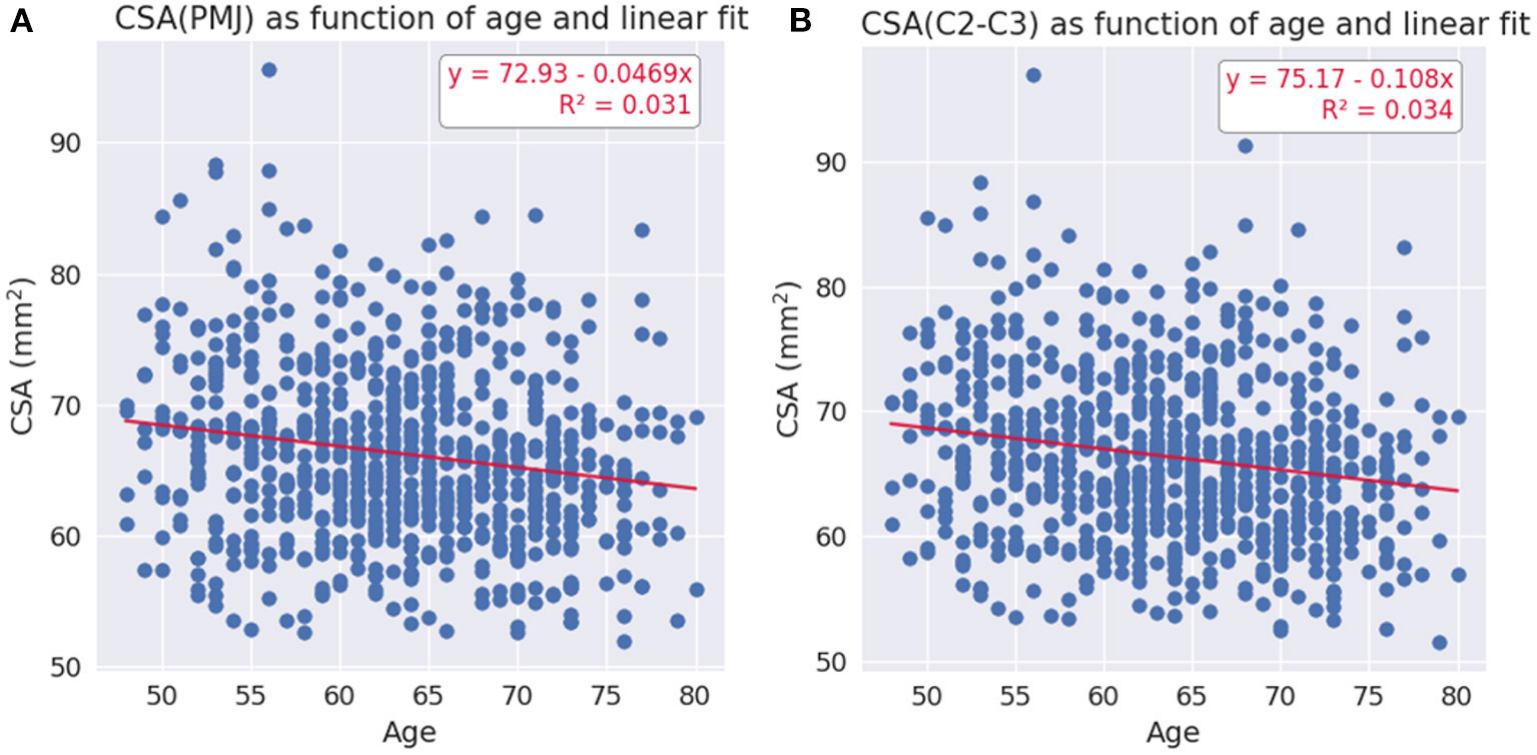
**(A)** Linear fit for CSA at 64 mm from the PMJ as a function of age. **(B)** Linear fit for CSA at C2–C3 disc as a function of age.

To explore the relationship between age and SC CSA, we calculated a linear and quadratic fit. The age of the participants ranged between 48 and 80 years old. The linear fits are presented at Figure 5. The equations for the quadratic fit are:


$$\begin{array}{l} \text{CSA}\left(\text{PMJ}\right):y\;=\;72.93\;-0.0469\cdot x\;-\;0.000907\cdot x^{2};\\ R^{2}\;=\;0.031\\ \text{CSA}\left(\text{C2–C3}\right):y\;=\;75.17\;-0.1076\cdot x\;-0.000475\cdot x^{2};\\ R^{2}\;=\;0.034 \end{array}$$

The constant and the linear coefficient are the same for both linear and quadratic fits per CSA methods (PMJ and C2–C3). The quadratic coefficient is very small for both methods (**CSA(PMJ**): 9.07 × 10^−4^; **CSA(C2–C3)**: 4.75 × 10^−4^). There is almost no quadratic trend in both cases.

#### Multilinear regression

Based on the *Pearson's* correlation analysis presented in Table 1, the following parameters were input in the stepwise linear regression: sex, height, weight, age, brain volume, ventricular CSF volume, and thalamus volume. Since brain WM volume, GM volume and brain volume have a very strong correlation (0.94 and 0.96), brain WM volume and GM volume were not included in the model to avoid collinearity.

The stepwise method yielded a model including brain volume and thalamus volume for CSA(PMJ) and CSA(C2–C3). The resulting model is shown in Table 2 for CSA at 64 mm from the PMJ and at Table 3 for CSA at C2–C3 disc. Adjusted *R*^2^ is 0.265 for CSA(PMJ) and 0.271 for CSA(C2–C3). Both models show a significant association with CSA (*p*-value < 0.0001).

**Table 2:** Multilinear regression analysis for SC CSA(PMJ) (*N* = 804 participants).

***R^2^***	***R^2^*** ** adj**	***F***	***p*** **-value**	**AIC**
**0.267**	**0.265**	**145.7**	**1.077e−54**	**5,093**
	**Coeff**	***t***	***p*** **-value**	
**Const**	27.18	11.634	5.154e−29	
**Thalamus volume**	1.99e−03	8.344	3.131e−16	
**Brain volume**	7.56e−06	2.407	0.016	

**Table 3:** Multilinear regression analysis for SC CSA(C2–C3) (*N* = 804 participants).

***R^2^***	***R^2^*** ** adj**	***F***	***p*** **-value**	**AIC**
**0.267**	**0.269**	**148.7**	**1.18e−55**	**5,072**
	**Coeff**	***t***	***p*** **-value**	
**Const**	27.49	11.924	2.737e−30	
**Thalamus volume**	0.002	8.498	9.373e−17	
**Brain volume**	7.30e−06	2.355	0.019	

### Normalization

#### Brain and thalamus volumes

The presented model's coefficients led to the following normalization equation with thalamus volume and brain volume and their respective mean for CSA at 64 mm from the PMJ and at C2–C3 disc. Normalized CSA(PMJ) had a mean and STD of 66.26 ± 5.72 mm^2^ and for CSA(C2–C3), 66.40 ± 5.64 mm^2^.

***CSA(PMJ):***


$$\begin{array}{l} CSA_{norm}^{i}=CSA_{meas}^{i}\;+1.986\cdot 10^{-3}\cdot (15266-X_{TV}^{\;i})\\ \;+7.56\;\cdot 10^{-6}\cdot (1156171-X_{BV}^{\;i})\\ \;z\text{-score}=\frac{CSA_{norm}-66.26}{5.72} \end{array}$$

***CSA(C2–C3):***


$$\begin{array}{l} \;\begin{array}{l} CSA_{norm}^{i}=CSA_{meas}^{i}\;+0.002\cdot (15266-X_{TV}^{\;i})\\ \;+\;7.30\cdot 10^{-6}\cdot (1156171-X_{BV}^{\;i}) \end{array}\\ \begin{array}{l} TV:\;thalamus\;volume(mm^{3});\;BV:\;brain\;volume\;(mm^{3}) \end{array}\\ \;\begin{array}{l} z\text{-score}=\frac{CSA_{norm}-66.40}{5.64} \end{array} \end{array}$$

With the CSA at 64 mm from the PMJ normalization, COV went from 10.09 ($CSA_{meas}^{i}$) to 8.64% $\left(CSA_{norm}^{i}\right)$, a reduction of 14.37%. With the CSA at C2–C3 disc normalization, COV went from 9.96 ($CSA_{meas}^{i}$) to 8.51% $CSA_{norm}^{i}$) a reduction of 14.61%.

#### Brain and thalamus volumes and sex interaction with brain volume

**Figure 6:**
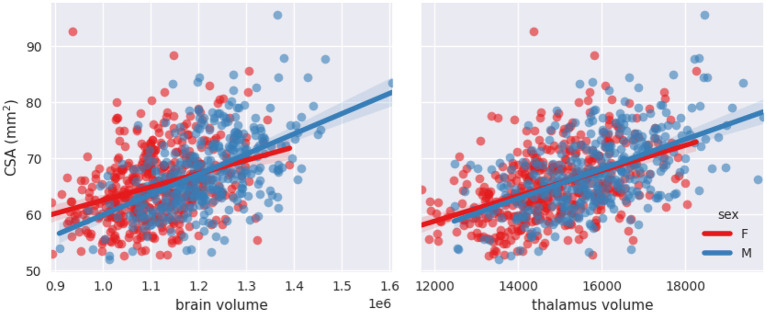
Scatterplots of CSA(PMJ) as a function of brain volume and thalamus volume separated for sex with linear fit.

**Figure 7:**
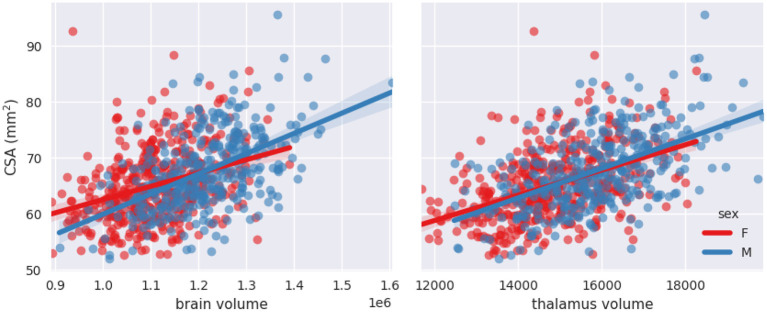
Scatterplots of CSA(C2–C3) as a function of brain volume and thalamus volume separated for sex with linear fit.

Figures 6, 7 show scatterplots of CSA at 64 mm from the PMJ and at the C2–C3 disc with both predictors (brain volume and thalamus volume) separated for sex. Qualitatively, we observe that the slopes for female and male are different with the brain volume predictor, however the slopes are closer with the thalamus volume predictor. To quantitatively validate if the interaction coefficient is significant in the model, we computed the interaction of both predictors with sex. The interaction coefficient for brain volume was significant (***CSA(PMJ)***: *p*-value = 0.006; ***CSA(C2–C3)***: *p*-value = 0.005) and sex was also significant when adding the interaction parameter (***CSA(PMJ)***: *p*-value = 0.005; ***CSA(C2–C3)***: *p*-value = 0.004). For thalamus volume, the interaction coefficient was not significant (***CSA(PMJ)***: *p*-value = 0.227; ***CSA(C2–C3)***: *p*-value = 0.218) neither was sex (***CSA(PMJ)***: *p*-value = 0.227; ***CSA(C2–C3)***: *p*-value = 0.213). The interaction of brain volume and sex was therefore added to the previous model since it has a significant effect. The following equation presents the corresponding normalization equation: ***CSA(PMJ):***


$$\begin{array}{l} \begin{array}{l} CSA_{norm}^{i}=CSA_{meas}^{i}\;+1.98\cdot 10^{-3}\cdot (15266-X_{TV}^{\;i}{}_{\;})\\ \;+\;2.45\;\cdot 10^{-6}\cdot (1156171-X_{BV}^{i})\\ \;-\;15\cdot (0.437-X_{sex}^{i})\\ \;+\;1.26\;\cdot 10^{-5}\cdot (530335-\;X_{sex}^{i}\cdot X_{BV}^{\;i}) \end{array}\\ \begin{array}{lll} \;z\text{-score} & = & \frac{CSA_{norm}-66.26}{5.59} \end{array} \end{array}$$

***CSA(C2–C3):***


$$\begin{array}{l} \begin{array}{l} CSA_{norm}^{i}=CSA_{meas}^{i}\;+1.98\cdot 10^{-3}\cdot (15266-X_{TV}^{\;i}{}_{\;})\\ \;+\;2.49\;\cdot 10^{-6}\cdot (1156171-X_{BV}^{\;i})\\ \;-\;1.235\cdot (0.437-X_{sex}^{i})\\ \;+\;1.26\;\cdot 10^{-5}\;\cdot (530335-\;X_{sex}^{i}\cdot X_{BV}^{\;i}) \end{array}\\ \begin{array}{l} \;z\text{-score}=\frac{CSA_{norm}-66.40}{5.61} \end{array} \end{array}$$

The model was significant (*p*-value < 0.0001) for both CSA(PMJ) and CSA(C2–C3). Normalized CSA(PMJ) had a mean and STD of 66.26 ± 5.69 mm^2^ and for CSA(C2–C3), 66.40 ± 5.61 mm^2^. The COV of CSA(PMJ) went from 10.09 to 8.59%, a reduction of 14.85%. The COV of CSA(PMJ) went from 9.96 to 8.42%, a reduction of 15.13%. Adjusted *R*^2^ was 0.271 for CSA(PMJ) and 0.276 for CSA(C2–C3).

#### Brain volume and sex interaction

Since measuring thalamus volume is not always convenient, we also proposed a model without the thalamus volume as a predictor and kept only brain volume, sex, and the interaction. Normalized CSA(PMJ) had a mean and STD of 66.26 ± 5.94 mm^2^ and for CSA(C2–C3), 66.40 ± 5.86 mm^2^. The COV went from 10.09 to 8.96%, a reduction of 11.22% for CSA(PMJ) and the adjusted *R*^2^ was 0.209. The COV went from 9.96 to 8.88%, a reduction of 11.39% for CSA(C2–C3) and the adjusted *R*^2^ was 0.212. Both models were also significant (*p*-value < 0.0001). Even if this model is less performant than the one including the thalamus volume, we present it given that thalamus volume is not often measured in neuroimaging analysis pipelines or in the presence of thalamic atrophy (Rocca et al., 2010; Foo et al., 2017; Azevedo et al., 2018; Eshaghi et al., 2018; Schönecker et al., 2018; Finegan et al., 2020). The normalization model has the following equation:

***CSA(PMJ):***


$$\begin{array}{l} \begin{array}{l} CSA_{norm}^{i}=CSA_{meas}^{i}\;+\;2.37\cdot 10^{-5}\cdot (1156171-X_{BV}^{\;i})\\ \;-\;15\cdot \;(0.437\;-X_{sex}^{\;i}\;)\\ \;+\;1.24\;\cdot 10^{-5}\cdot (530335\;-X_{sex}^{i}\cdot X_{BV}^{\;i}) \end{array}\\ \begin{array}{l} \;z\text{-score}=\frac{CSA_{norm}-66.26}{5.94}\; \end{array} \end{array}$$

***CSA(C2–C3):***


$$\begin{array}{l} \begin{array}{l} CSA_{norm}^{i}=CSA_{meas}^{i}\;+\;2.38\cdot 10^{-5}\cdot (1156171-X_{BV}^{\;i})\\ \;-\;15.23\cdot \;(0.437\;-X_{sex}^{\;i}\;)\\ \;+\;1.25\;\cdot 10^{-5}\cdot (530335\;-X_{sex}^{i}\cdot X_{BV}^{\;i}) \end{array}\\ \begin{array}{l} \;z\text{-score}=\frac{CSA_{norm}-66.40}{5.86} \end{array} \end{array}$$

## Discussion

In this work, we quantified the contribution of various factors on inter-subject variability in cervical SC CSA measurements in 804 participants with a fully-automatic processing pipeline. We implemented a measurement method for SC CSA that uses the PMJ as opposed to the vertebral reference to overcome its limitations associated with the head position variability. Finally, we developed a normalization model which can reduce inter-subject variability by up to 14.85% for CSA(PMJ) or 15.13% for CSA(C2–C3).

### CSA results

We obtained mean CSA values of 66.2 and 66.4 mm^2^ for PMJ-based and vertebral-based CSA respectively, which is lower than what was reported in other studies (Papinutto et al., 2020; Solstrand Dahlberg et al., 2020; Kesenheimer et al., 2021). Lower values could be explained by various factors. Firstly, the population studied here is relatively older than in other published studies (range: 48–80, mean: 64). Secondly, the segmentation method has an impact on defining the boundary between the SC and surrounding CSF. SCT's sct_deepseg_sc is more conservative than other software in defining this border, which results in a smaller CSA (Weeda et al., 2019; Lukas et al., 2021). This is due to the segmentation algorithm: if we adjust the threshold for determining the boundary between the SC and the background (CSF), the produced segmentation becomes systematically scaled up/down depending on the threshold value. This translates to a systematic bias, similar to a calibration problem, which applies, in average, equally across data quality. Therefore, when it comes to using CSA values for clinical studies, it only adds an offset and does not affect the precision of the measure. Thirdly, acquisition parameters (which drive image contrast) also influence CSA values (Kearney et al., 2014; Papinutto and Henry, 2019; Cohen-Adad et al., 2021). Furthermore, gradient echo T1w acquisitions are prone to motion artifacts, which hamper the performance of SC segmentation. COV were 9.96 and 10.09% (C2–C3, PMJ), which is similar to what was observed in previous studies (Papinutto et al., 2020; Solstrand Dahlberg et al., 2020; Kesenheimer et al., 2021).

### PMJ-based CSA method

Regarding vertebral-based CSA and PMJ-based CSA, COVs are very similar (9.96% C2–C3, 10.09% PMJ) as for CSA values (see Figure 3). The CSA measures between the PMJ and C2–C3 disc reference were statistically different (*p*-value = 0.0002). This suggests that using the PMJ as a reference for CSA measurement is relevant to overcome the imprecise prediction of the spinal segments. This enforces the fact that vertebral levels are not a precise surrogate for spinal levels. In terms of variability, there is no clear conclusion if the PMJ-based method reduces the inter-subject variability, which is a relevant indicator in cross-sectional studies (e.g., used to assess the signature of a biomarker with different phenotypes). However, for longitudinal studies (i.e., intra-subject COV), given that head tilting might change across sessions, it is possible that a PMJ-based method is preferred. While no clear conclusion can be drawn from the PMJ-based method in comparison with the vertebral-based method in terms of inter-subject CSA variability, this study sets the foundations, which are hard to establish, to further investigate the relevance of a PMJ-based method since it is fully-automatic and suggests promising results for longitudinal studies.

Some limitations are associated with the PMJ-based CSA method. The use of the PMJ label to interpolate with the centerline is not the exact extrapolation of the centerline. SC curvature at the PMJ varies across individuals, which adds variability to the computed distance. PMJ label positioning across participants may also differ, also affecting the measured distance.

Moreover, the absolute distance from PMJ doesn't consider the fact that the SC length varies across individuals (Lang and Bartram, 1982; Boonpirak and Apinhasmit, 1994). Using a relative distance to predict the spinal segments from the PMJ could be relevant to overcome this limitation. Results from this study show a difference between SC CSA using a vertebral-based vs. PMJ-based reference, but not between the inter-subject variability. A comparison with the nerve rootlets is necessary to assess which method ensures proper prediction of the spinal segments; it will be the subject of further investigations.

Another question remains about the robustness of the PMJ-based CSA method in the presence of lesions in the brainstem and the spinal cord. The presence of lesions in the brainstem could lower the performance of the automated identification of the PMJ, which needs to be further assessed in pathological data. The other important aspect of the PMJ-based method is the spinal cord centerline, which is extracted from the spinal cord segmentation. In the presence of lesions, the automatic segmentation could also be less reliable, requiring manual correction. Future developments of spinal cord segmentation algorithms that are robust to the presence of lesions would address this limitation.

In the case of high atrophy rate in the brainstem, we expect that it is very unlikely that the PMJ won't be easily identifiable, considering it is the only required label in the brainstem region for this method. Additionally, we don't expect that the centerline extraction will be affected by lesions in the brainstem since it only relies on the spinal cord segmentation. However, a high atrophy rate in the spinal cord would affect the spinal cord segmentation. The centerline would lose some precision since it is extracted by computing the center of mass of each slice of the spinal cord segmentation. This could result in loss of precision in the computation of the distance from the PMJ in order to compute CSA, but further investigations are needed to establish the impact of lesions in the brainstem or spinal cord on the PMJ-based CSA method.

### Correlation with SC CSA

Our analysis shows a strong correlation between brain volume, brain WM volume, brain GM volume and thalamus volume with CSA, as previous studies have reported (Engl et al., 2013; Papinutto et al., 2015, 2020; Solstrand Dahlberg et al., 2020). The highest correlation was found with thalamus volume (*Pearson's r* = 0.51). Numerous ascending tracts project from the spinal cord to various thalamus nuclei (spinothalamic tract), which could explain a close correlation between CSA and thalamus volume compared to other brain structures (Giesler and Willis, 1981; Hodge and Apkarian, 1990; Torrico and Munakomi, 2022). No significant correlation was found with ventricular CSF volume. Correlations with height and weight are low.

It would have been interesting to explore other potential regressors explaining SC CSA, such as other deep gray matter structures, as well as brainstem structures (e.g., pons, medulla) due to their closeness to the cervical SC. These additional regressors would help elucidate if the strong correlation between the thalamus volume and SC CSA is unique to the thalamus or if it is also found in other structures.

We found a statistical difference between SC CSA between male and female; females have a significantly smaller CSA than males as previous studies have shown (Papinutto et al., 2015, 2020; Solstrand Dahlberg et al., 2020).

Regarding the effect of age on SC CSA, we found a decrease of CSA with age. Linear and quadratic fit gave very similar results (**CSA(PMJ):** *R*^2^ = 0.031 and **CSA(C2–C3):** *R*^2^ = 0.034 for both fits). Since the age range of the participants goes from 48 to 80 years old, it is not surprising that a linear fit is also adequate, in comparison with results reported by others (Papinutto et al., 2020; Kesenheimer et al., 2021). Since CSA peaks around 45 years old (Papinutto et al., 2020; Kesenheimer et al., 2021), CSA values for the age range of this study are decreasing with age as we observed (see Figure 5).

### Normalization methods

Stepwise linear regression led to thalamus volume and brain volume as predictors for SC CSA. We introduce for the first time thalamus volume as a predictor for normalization of SC CSA. Thalamus volume presented a high correlation with brain volume (*Pearson's r* = 0.79), hence some collinearity between both predictors may reduce the statistical power in the obtained model. However, we decided to keep both variables in order to preserve some potentially useful interaction measures. This model significantly reduced inter-subject variability; COV went down from 10.09 to 8.64%, which represents a reduction of 14.37% for CSA(PMJ). COV went down from 9.96 to 8.51%, which represents a reduction of 14.61% for CSA(C2–C3). Other parameters were not significant since they were excluded during the stepwise model (*p*-value > 0.05). Sex alone was not a significant predictor (*p*-value > 0.05) even if there is a significant difference between male and female CSA. Note the strong correlation between sex and thalamus volume (*Pearson's r* = 0.36) and between sex and brain volume (Pearson's *r* = 0.49). When adding the interaction between sex and brain volume, sex and the interaction became significant. The effect of brain volume on SC CSA varies between male and female as we can observe in Figures 6, 7. The interaction between thalamus volume and sex wasn't significant. The model including brain volume, thalamus volume, sex and sex/brain volume interaction led to a COV of 8.59%, a reduction of 14.85 % for CSA(PMJ) and for CSA(C2–C3), a COV of 8.42%, a reduction of 15.13%. Including sex and brain volume interaction led to the best COV reduction. To our best knowledge, interaction of sex and brain volume was never considered in previous normalization models, only the fixed effect of sex on CSA was included. These findings reveal the importance to consider that factors can vary differently for males and females. We also proposed a model without the thalamus volume, given the difficulty to measure it (it requires the proper anatomical sequence with sufficient contrast and resolution) and/or in the case of abnormal thalamic atrophy, which could happen in various pathological conditions. This model reduced CSA variability less than when including thalamus volume [11.22% of reduction for CSA(PMJ) vs. 11.39% for CSA(C2–C3)]. The combination of thalamus volume, brain volume and sex better explains CSA variability.

Even if CSA at 64 mm from the PMJ and at C2–C3 disc differed significantly, the normalization analysis and obtained models did not vary and the coefficients of the models were very similar.

We obtained a smaller reduction than other models presented in previous studies (Papinutto et al., 2020; Kesenheimer et al., 2021). Kesenheimer et al. (2021) obtained a reduction of COV of 23.7% using sex, brain WM volume and SC canal area, Papinutto et al. (2020) obtained a reduction of 17.74% using Vscale and axial-canal product. It is important to consider that the predictors of the normalization methods were different, mainly regarding metrics related to the SC canal which could explain the smaller reduction of COV obtained with our model [CSA(PMJ): 14.85% or CSA(C2–C3): 15.13%]. We did not include SC canal metrics in our analysis because of the lack of automated methods for robust SC canal segmentation combined with the large number of participants in this study. Also, the size of the cohort is larger than in the previously mentioned works, *N* = 60 for (Kesenheimer et al., 2021) and *N* = 129 for (Papinutto et al., 2020), which impacts the distribution and coverage of the data of the participants and affects the normalization method.

Age was not a significant predictor for SC CSA. Trends for CSA and brain volume for the age range of our study are very similar. The effect may differ for younger people since brain volume decreases linearly with age while CSA increases until about 45 years old and decreases afterward (Papinutto et al., 2020).

We have to consider the fact that older people may be more subject to motion in the MRI than younger people (discomfort, difficulty breathing) resulting in a bias in the measured CSA (blurring, motion artifact ghosting). Further investigations are needed to validate if the model can expand to ages not included in this study.

The normalization model was generated from T1w data with a specific protocol. Subsequent studies should assess whether the model is adequate for other acquisition parameters and contrasts. It is known that the output CSA varies for different acquisition protocols (Cohen-Adad et al., 2021). However, since there is a direct relationship between CSA values from different contrasts, there would be a systematic offset in the produced CSA. Since the model is linear, it should hold for different contrasts.

It is important to note that the choice of processing software package (and its version) used to compute the brain morphometrics affects the measured brain and thalamus volumes, and hence the proposed normalization model. The proposed normalization model would then require data to be processed using the same processing software as that used for the UK Biobank analysis pipeline (Alfaro-Almagro et al., 2018).

The model was developed from healthy participants; the question remains if it would be applicable to patients with neurodegenerative diseases such as MS. Thalamic atrophy is common in neurodegenerative diseases such as MS (Rocca et al., 2010; Foo et al., 2017; Azevedo et al., 2018; Eshaghi et al., 2018; Schönecker et al., 2018; Finegan et al., 2020). In the case of such atrophy, one should refrain from normalizing CSA with thalamus volume since the normalization would be biased. This is why we proposed a model without the thalamus volume. Also, brain volume changes have been associated with atrophy for various neurodegenerative diseases. A normalization model including brain volume may not be generalizable for those patients. As done in other studies (Kesenheimer et al., 2021), intracranial volume could be a relevant substitute for brain volume since it is not affected by neurodegenerative diseases. Including sex interaction here will also be important and can improve the normalization model. Further studies could include intracranial volume in the normalization model.

Furthermore, other confounding factors could possibly affect image acquisitions for pathological patients. Severe motor disability could induce some breathing difficulties which induces considerable motion artifacts, thus SC segmentation and CSA bias.

### SCT normalization feature

We made available the obtained normalization model in the open-source software SCT within sct_process_segmentation. Since thalamus volume may not be available in all SC MRI studies, we made it possible for the user to normalize CSA values without thalamus volume. Even if the best model was obtained with thalamus volume, brain volume, sex and sex interaction between brain volume and sex, normalizing SC CSA without thalamus volume could still reduce CSA variability. The normalization feature can be used by adding the option -*normalize* followed by the predictors and their corresponding values. For more information on usage, refer to: https://spinalcordtoolbox.com/user_section/command-line.html#sct-process-segmentation.

## Conclusions

This study features an analysis of factors contributing to SC CSA variability at a larger scale than what was done previously to our best knowledge using an automatic processing pipeline. We introduced a new reference in the context of CSA measurements based on a neurological reference (PMJ) to overcome vertebral reference limitations (neck flexion and extension) which sets ground for further investigation regarding the prediction of spinal segments and cervical CSA studies. We computed over a large cohort of participants SC CSA at 64 mm from the PMJ on T1w scans from the UK Biobank database. The pipeline is based on regular brain MRI scans, making it of interest for a broad range of study types, including clinical studies. No significant age trend was found while SC CSA was significantly different for males and females. We present an effective normalization model including thalamus volume, brain volume, sex and sex/brain volume interaction readily usable in SCT. The most relevant factors to explain SC CSA variability are related to the brain; these findings show the importance of having a brain MRI acquisition in SC studies/research. Reducing inter-subject variability could improve comparison between CSA measures to increase its sensitivity and specificity to better assess pathology-related changes.

## Data availability statement

UK Biobank data are available through the following procedure https://www.ukbiobank.ac.uk/enable-your-research. Requests to access these datasets should be directed to https://www.ukbiobank.ac.uk/enable-your-research.

## Author contributions

SB: conceptualization, data curation, investigation, methodology, software, validation, and writing—original draft. JC-A: conceptualization, methodology, supervision, software, validation, writing—original draft, writing—review, and editing. Both authors contributed to the article and approved the submitted version.

## Funding

This study was funded by the Canada Research Chair in Quantitative Magnetic Resonance Imaging [950-230815], the Canadian Institute of Health Research [CIHR FDN-143263], the Canada Foundation for Innovation [32454 and 34824], the Fonds de Recherche du Québec—Santé [28826], the Natural Sciences and Engineering Research Council of Canada [RGPIN-2019-07244], the Canada First Research Excellence Fund (IVADO and TransMedTech), the Courtois NeuroMod project, the Quebec BioImaging Network [5886 and 35450], the Mila—Tech Transfer Funding Program, and the Spinal Research and Wings for Life (INSPIRED project).

## Conflict of interest

The authors declare that the research was conducted in the absence of any commercial or financial relationships that could be construed as a potential conflict of interest.

## Publisher's note

All claims expressed in this article are solely those of the authors and do not necessarily represent those of their affiliated organizations, or those of the publisher, the editors and the reviewers. Any product that may be evaluated in this article, or claim that may be made by its manufacturer, is not guaranteed or endorsed by the publisher.

## Footnotes

^1^ https://biobank.ndph.ox.ac.uk/showcase/label.cgi?id=2406

^2^ https://github.com/spine-generic/spine-generic/releases/tag/v2.6

^3^ https://github.com/neuropoly/spinalcordtoolbox/releases/tag/5.4

^4^ https://github.com/sct-pipeline/ukbiobank-spinalcord-csa/tree/v1.1#cord-csa-on-uk-biobank-brain-mri-databas

## Abbreviations

BMI: body mass index
COV: coefficient of variation
CSA: cross-sectional area
CSF: cerebrospinal fluid
GM: gray matter
MS: multiple sclerosis
PMJ: pontomedullary junction
SC: spinal cord
SCT: spinal cord toolbox
STD: standard deviation
Vscale: scaling factor for brain normalization due to differences in head size (SIENAX)
WM: white matter.
