# Supplementary material for: Automatic measure and normalization of spinal cord cross-sectional area using the pontomedullary junction
Source: Front Neuroimaging. 2022 Nov 2;1:1031253. doi: 10.3389/fnimg.2022.1031253 (PMC10406309; doi:10.3389/fnimg.2022.1031253)
Supplement: Supplementary file 1 [file Data_Sheet_1.docx]

Supplementary Material

# Supplementary Figures and Tables

## Supplementary Figures


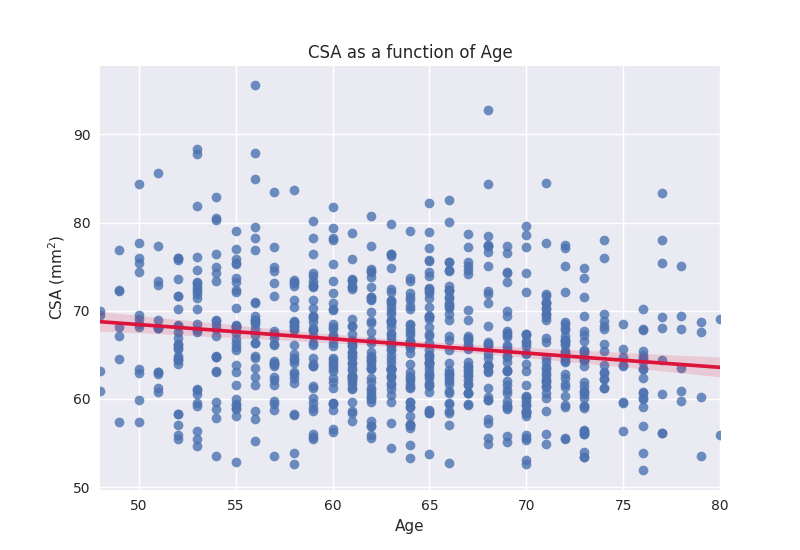


**Figure S1.** Scatterplot of CSA at 64 mm from the PMJ as a function of age.


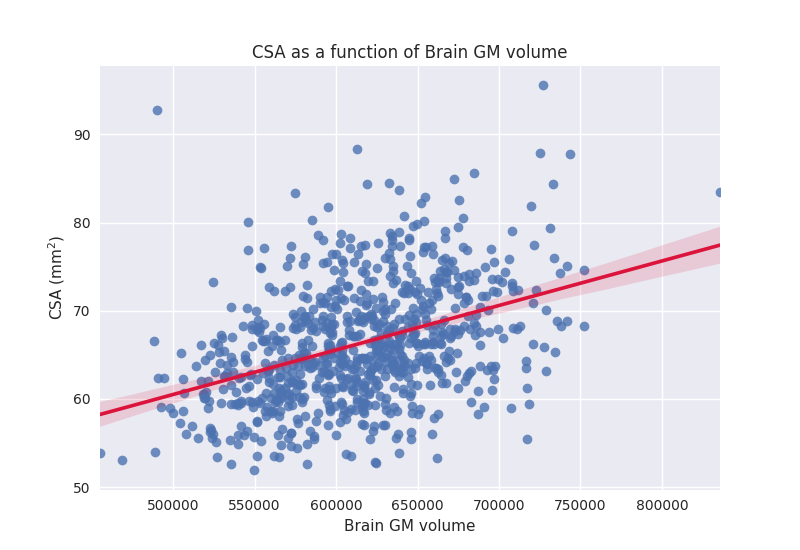
[Figure S](file:///C:\Users\sb199\Downloads\csa_ukbiobank_manuscript_r4%20(1).docx#fisup_scatterplot_brain_gm_vol)2. Scatterplot of CSA at 64 mm from the PMJ as a function of brain GM volume.

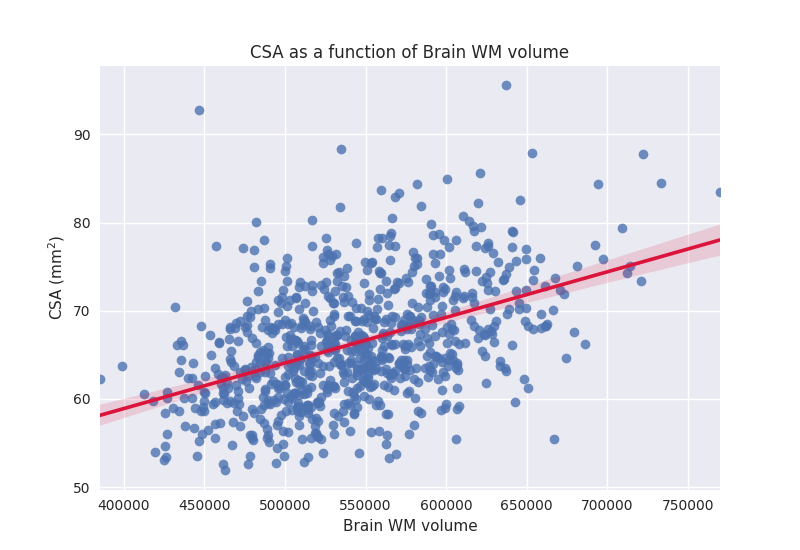
[Figure S](file:///C:\Users\sb199\Downloads\csa_ukbiobank_manuscript_r4%20(1).docx#fisup_scatterplot_brain_wm_vol)3. Scatterplot of CSA at 64 mm from the PMJ as a function of brain volume WM volume.

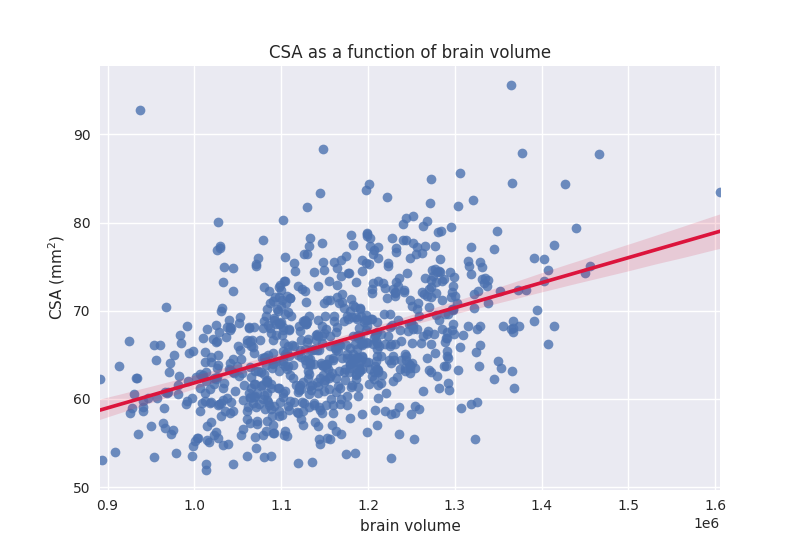
[Figure S](file:///C:\Users\sb199\Downloads\csa_ukbiobank_manuscript_r4%20(1).docx#fisup_scatterplot_brain_vol)4. Scatterplot of CSA at 64 mm from the PMJ as a function of brain volume.

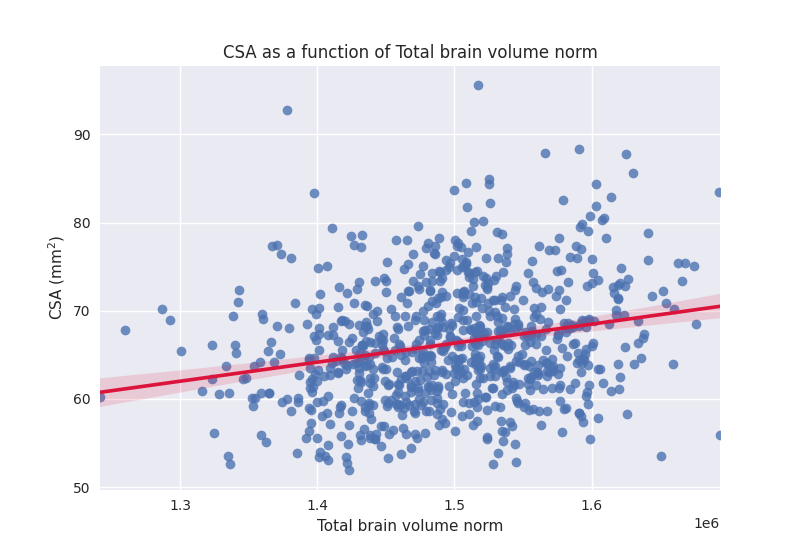
[Figure S](file:///C:\Users\sb199\Downloads\csa_ukbiobank_manuscript_r4%20(1).docx#fisup_scatterplot_brain_vol_norm)5. Scatterplot of CSA at 64 mm from the PMJ as a function of brain volume normalized for head size.


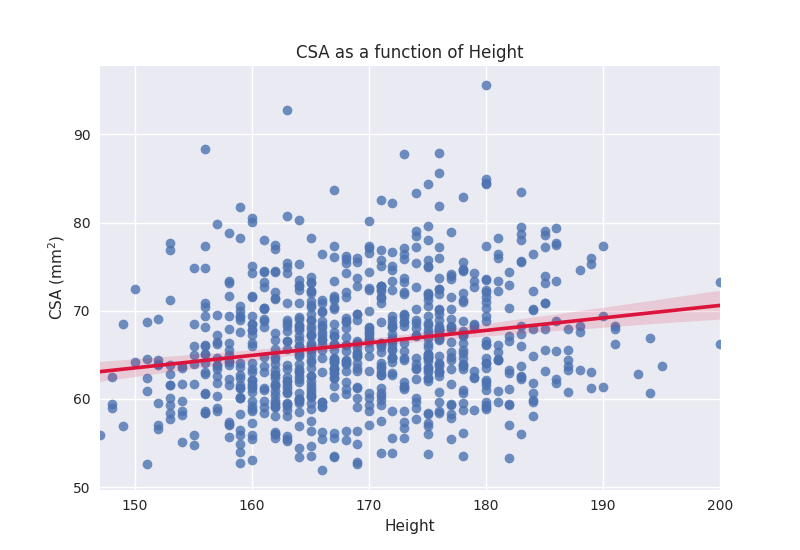
[Figure S](file:///C:\Users\sb199\Downloads\csa_ukbiobank_manuscript_r4%20(1).docx#fisup_scatterplot_height)6. Scatterplot of CSA at 64 mm from the PMJ as a function of height.

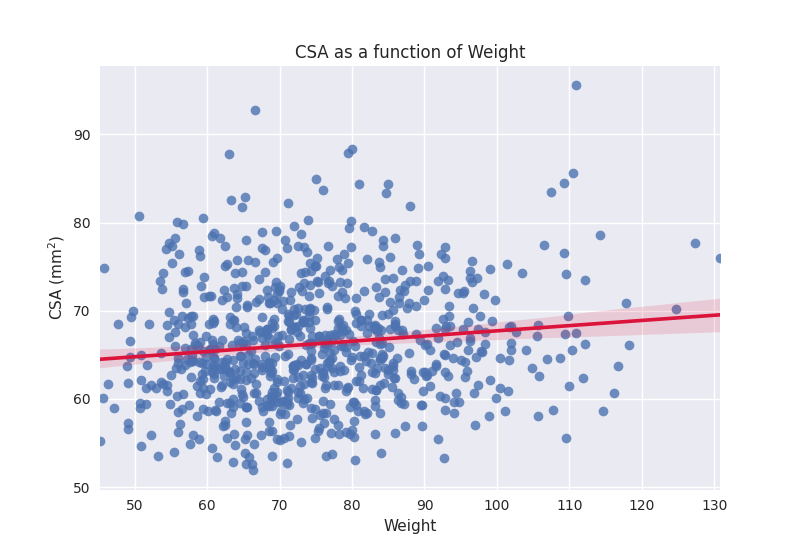
[Figure S](file:///C:\Users\sb199\Downloads\csa_ukbiobank_manuscript_r4%20(1).docx#fisup_scatterplot_weight)7. Scatterplot of CSA at 64 mm from the PMJ as a function of weight.


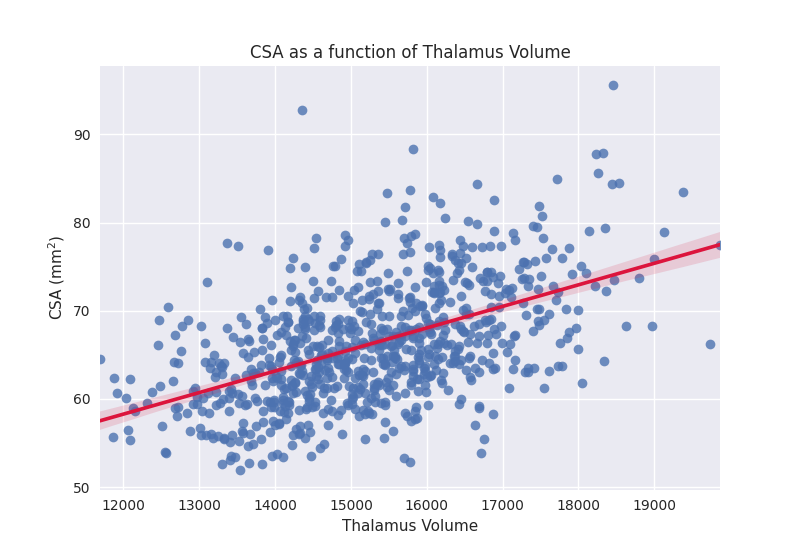
[Figure S](file:///C:\Users\sb199\Downloads\csa_ukbiobank_manuscript_r4%20(1).docx#fisup_scatterplot_thalamus)8. Scatterplot of CSA at 64 mm from the PMJ as a function of thalamus volume.

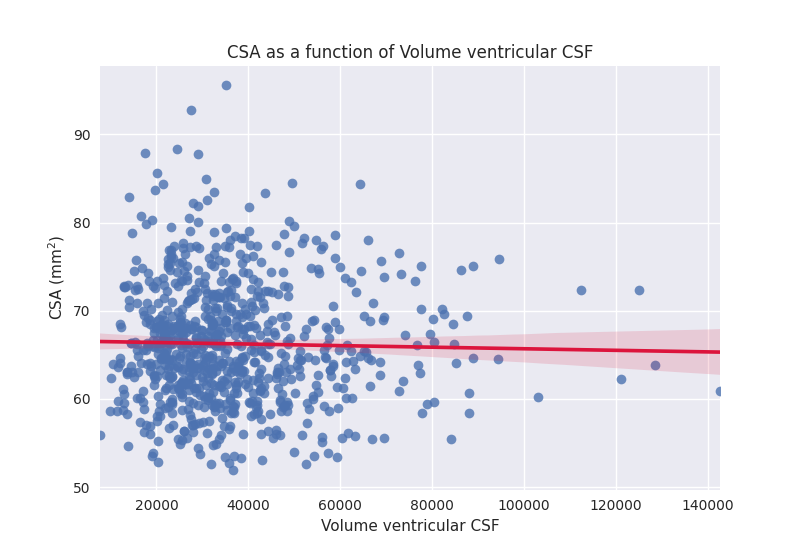
[Figure S](file:///C:\Users\sb199\Downloads\csa_ukbiobank_manuscript_r4%20(1).docx#fisup_scatterplot_csf_vol)9. Scatterplot of CSA at 64 mm from the PMJ as a function of ventricular CSF volume.


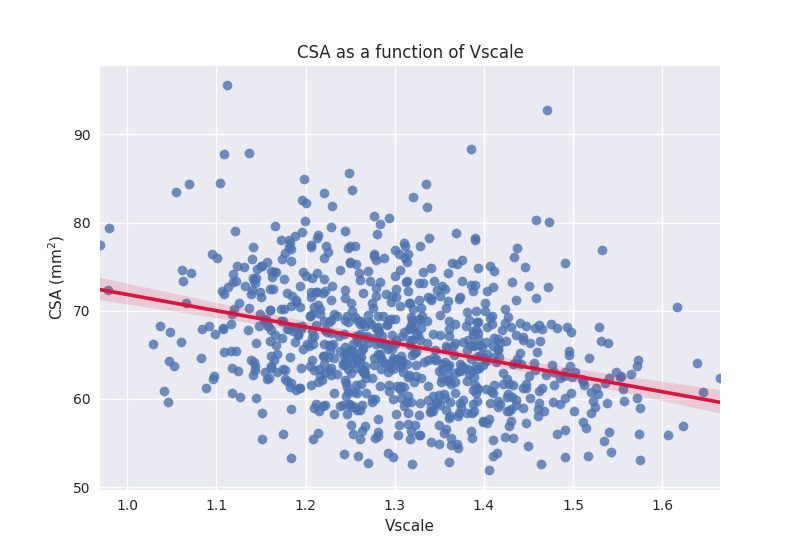
[Figure S10](file:///C:\Users\sb199\Downloads\csa_ukbiobank_manuscript_r4%20(1).docx#fisup_scatterplot_vsacle). Scatterplot of CSA at 64 mm from the PMJ as a function of v-scale.
